# Enhancing plant growth in biofertilizer-amended soil through nitrogen-transforming microbial communities

**DOI:** 10.3389/fpls.2023.1259853

**Published:** 2023-11-14

**Authors:** Liangzhi Li, Zhengrong Hu, Ge Tan, Jianqiang Fan, Yiqiang Chen, Yansong Xiao, Shaolong Wu, Qiqi Zhi, Tianbo Liu, Huaqun Yin, Qianjun Tang

**Affiliations:** ^1^ College of Plant Protection, Hunan Agricultural University, Changsha, China; ^2^ School of Minerals Processing and Bioengineering, Central South University, Changsha, China; ^3^ Key Laboratory of Biometallurgy of Ministry of Education, Central South University, Changsha, China; ^4^ Hunan Tobacco Research Institute, Changsha, China; ^5^ China Tobacco Hunan Industrial Co., Ltd., Changsha, China; ^6^ Technology Center, China Tobacco Fujian Industrial Co., Ltd., Xiamen, Fujian, China; ^7^ Chenzhou Tobacco Company of Hunan Province, Chenzhou, China

**Keywords:** biofertilizer, binning, metagenomics, microbial interaction, keystone taxa, soil

## Abstract

Biofertilizers have immense potential for enhancing agricultural productivity. However, there is still a need for clarification regarding the specific mechanisms through which these biofertilizers improve soil properties and stimulate plant growth. In this research, a bacterial agent was utilized to enhance plant growth and investigate the microbial modulation mechanism of soil nutrient turnover using metagenomic technology. The results demonstrated a significant increase in soil fast-acting nitrogen (by 46.7%) and fast-acting phosphorus (by 88.6%) upon application of the bacterial agent. This finding suggests that stimulated soil microbes contribute to enhanced nutrient transformation, ultimately leading to improved plant growth. Furthermore, the application of the bacterial agent had a notable impact on the accumulation of key genes involved in nitrogen cycling. Notably, it enhanced nitrification genes (*amo, hao*, and *nar*), while denitrification genes (*nir* and *nor*) showed a slight decrease. This indicates that ammonium oxidation may be the primary pathway for increasing fast-acting nitrogen in soils. Additionally, the bacterial agent influenced the composition and functional structure of the soil microbial community. Moreover, the metagenome-assembled genomes (MAGs) obtained from the soil microbial communities exhibited complementary metabolic processes, suggesting mutual nutrient exchange. These MAGs contained widely distributed and highly abundant genes encoding plant growth promotion (PGP) traits. These findings emphasize how soil microbial communities can enhance vegetation growth by increasing nutrient availability and regulating plant hormone production. This effect can be further enhanced by introducing inoculated microbial agents. In conclusion, this study provides novel insights into the mechanisms underlying the beneficial effects of biofertilizers on soil properties and plant growth. The significant increase in nutrient availability, modulation of key genes involved in nitrogen cycling, and the presence of MAGs encoding PGP traits highlight the potential of biofertilizers to improve agricultural practices. These findings have important implications for enhancing agricultural sustainability and productivity, with positive societal and environmental impacts.

## 1 Introduction

Nitrogen plays a vital role in plant growth (Chen et al., 2021), and its uptake by plants is primarily influenced by nitrogen availability. Recent studies have highlighted the critical contribution of soil microbes in determining soil nitrogen availability and crop nitrogen uptake. Soil microbes actively participate in the biogeochemical nitrogen cycle and facilitate plant nitrogen acquisition in agroecosystems (Kuypers et al., 2018). Plants are capable of assimilating nitrogen in various forms, including ammonium, nitrate, and small molecule organic nitrogen. The transformation of nitrogen is mediated by soil microbes. Gaseous nitrogen undergoes fixation to ammonia, which is subsequently utilized by plants and microbes to produce organic nitrogen compounds like amino acids (Coruzzi, 2003). Ammonium can be released through the breakdown of organic matter and subsequently oxidized to nitrate by nitrification (1), eventually being converted to N_2_ through denitrification (2) or anaerobic ammonia oxidation (3) (Kuypers et al., 2018). Moreover, nitrate and nitrite can be reduced to ammonium through allotropic reduction (4) (Kuypers et al., 2018):

1. NH_4_^+^ → NO_2_^-^→ NO_3_^-^
2. NO_3_^-^→ NO_2_^-^→ NO → N_2_O → N_2_
3. NH_4_^+^ + NO_2_^-^→ N_2_
4. NO_3_^-^→ NO_2_^-^→ NH_4_^+^

Therefore, fluctuations in nitrogen transformation rates are typically associated with changes in the activities, compositions, and/or abundance of the corresponding microbial communities (Kuypers et al., 2018).

The use of microorganism-based biofertilizers is a significant technique already being employed to enhance plant growth under stressful conditions and minimize nitrogen loss (Vassilev et al., 2015; Allsup et al., 2023). This form of fertilizer is considered an environmentally friendly alternative to chemical fertilizers due to its ability to improve soil structure, restore soil fertility, maintain microbial balance in the rhizosphere, and degrade harmful chemicals (Wang et al., 2018). Unlike synthetic fertilizers, microbial agent-based biofertilizers can also reduce ammonia volatilization by inhibiting the conversion of nitrogen fertilizers into NH_4_^+^-N and promoting the nitrification process, thereby increasing the utilization of NH_4_^+^-N (Wang et al., 2018). Certain microbial agent-based biofertilizers, such as *Bacillus amyloliquefaciens* and *B. subtilis*, have shown significant potential in suppressing ammonia volatilization by 68% and 44%, respectively (Sun et al., 2020a; Xue et al., 2021). Additionally, viable and nonviable *Trichoderma viride* biofertilizers have been found to reduce ammonia loss by 42.21% and 32.42%, respectively (Wang et al., 2018). Moreover, biofertilizers can mitigate increases in soil pH that often result from excessive use of urea-based fertilizers (Wang et al., 2018). Additionally, biofertilizers enhance the population of nitrifying bacteria in the soil, facilitating the conversion of NH_4_^+^-N to NO_3_^–^N and reducing the release of nitrogen through ammonia volatilization (Wang et al., 2018). In recent years, microbial biofertilizers have emerged as a growing area of research in the field of nutrition modulation technologies. However, the production of biofertilizers with high-efficiency nutrient modulation capabilities and rapid environmental adaptation still faces challenges due to the demanding environmental requirements of microbes (Vassilev et al., 2015).

In this study, we developed a microbial agent with the aim of enhancing soil nutrient availability and improving plant growth. Our objective was to investigate the mechanisms through which the microbial agent enhances plant nitrogen uptake efficiency. We utilized metagenomics technology to identify the key microorganisms and functional genes that influence nitrogen uptake efficiency. The study had three specific aims: 1) addressing the problems caused by excessive fertilizer application, such as poor soil physical characteristics and low crop quality; 2) increasing soil nutrient availability; and 3) promoting plant growth.

## 2 Materials and methods

### 2.1 Assessment of physiochemical properties

Soil samples were collected from an experimental field cultivating *Nicotiana tabacum* at Hunan Agricultural University in Hunan Province, China, for the purpose of this study. Functional microorganisms with capacities for ammonia oxidation, denitrification, and nitrogen fixation were combined based on their nutrient types and oxygen requirements. The treatment group received a functional bacterial agent at a concentration of 2 × 10^7^ CFU/g, while the control group received an equal amount of sterilized functional bacterial agent. Soil and plant samples were collected at 30-day intervals after adding the bacterial agent, resulting in a total of 18 samples with triplicates.

The entire plants were uprooted and the roots were detached. Loosely attached soil was removed from the root system, and the remaining soil on the roots was brushed off. The soil adhering to the roots was considered as rhizosphere soil and labeled for subsequent bacterial community analysis and high-throughput sequencing. Plant growth traits such as primary root length, root maximum length, plant height, number of seminal roots, number of crown roots, number of leaves, shoot fresh weight, seminal root fresh weight, primary root fresh weight, crown root fresh weight, total root fresh weight, total plant fresh weight, root biomass, shoot biomass, root/shoot ratio, and total plant biomass were manually assessed.

Samples of the soil were collected from each stage in triplicate (n = 18). The soil in each pot was thoroughly mixed after removing the plants and debris. Multiple soil samples were taken from these pots, combined, and divided into two portions. One portion of the soil sample was sieved through a 2 mm aperture and stored at 4°C for future physiochemical property measurements. The remaining portion of the sample was placed in liquid nitrogen and stored at -80°C. The pH of the soil was measured using a conventional pH meter and a soil suspension (1:2.5 w/v), following the standard procedure specified in NY/T 1377-2007 (Chinese Standard). Clean water was heated, cooled, and vigorously mixed with soil samples for 10 minutes, and then allowed to settle for 1 hour. The concentrations of total nitrogen (TN), readily available nitrogen (AN), total phosphorus (TP), readily available phosphorus (AP), total potassium (TK), and readily available potassium (AK) in the soil were determined using the Kjeldahl method (Sáez-Plaza et al., 2013) for nitrogen, the KCl extraction method for nitrogen, the ClO_4_-H_2_SO_4_-molybdenum-antimony resistance colorimetric method for phosphorus, the NaHCO_3_ extraction-molybdenum-antimony resistance colorimetric method for phosphorus (Wieczorek et al., 2022), the NaOH fusion-flame photometry for potassium, and the NH_4_OAc extraction-flame photometry for potassium (Grewal et al., 2017), respectively.

### 2.2 DNA extraction and sequencing analysis

Frozen soil samples (0.25 g) were thawed on ice, and the DNA was extracted with the DNeasy PowerSoil Kit (Qiagen, Germany) according to the manufacturer’s protocol. The metagenomes were sequenced on an Illumina MiSeq at the Novogene Co., Ltd. (Hangzhou, China). The raw reads were trimmed and quality-controlled using Trimmomatic v.0.38 (minimum read length 20 and average base quality 20 in 4-base sliding windows) (Bolger et al., 2014), implementing the recommended parameters. The samples have generated a total of 180 GB raw data (n=18). The clean reads were assembled using MEGAHIT v1.0 (Li et al., 2015) (-k-min 21, –k-max 191, –min-contig-len 500). Accurate species abundance re-estimation was calculated using Bayesian Re-estimation of Abundance with KrakEN v2.0.9 and BRACKEN v2.6.0 using the default KRAKEN database (Lu & Salzberg, 2020) for all metagenomes. Resampling (1,000 permutations) of the taxonomic and functional abundance profiles and downstream analyses of the sequencing libraries were conducted in R v3.6 (https://www.r-project.org) using the package “phyloseq” (https://joey711.github.io/phyloseq/), “vegan” (https://www.rdocumentation.org/packages/vegan) and “plspm” (https://plspm.readthedocs.io/en/latest/) following corresponding instructions. Partial least-squares discriminant analysis (PLS-DA) was used to visualize discrimination among samples. This distance matrix was further analyzed using analysis of similarity (ANOSIM) in the R package vegan to analyze the differences between sample groups, following the instructions at https://www.rdocumentation.org/packages/vegan/versions/2.6-4/topics/anosim. The Statistical Analysis of Metagenomic Profiles (STAMP) software v2.1.3 (https://beikolab.cs.dal.ca/software/STAMP) was used to analyze statistically significant differences in the abundance of microbial taxa between control and treatment groups. Redundancy analysis (RDA) was used to illustrate the relative impacts of the geochemical parameters on the microbial community beta diversity using the vegan package, following the instructions at https://programmer.ink/think/r-redundancy-analysis-rda-ggplot2.html. The co-occurrence networks of both microbial interactions were calculated using Spearman’s correlation (ρ) > |0.8| and p < 0.05 in the CoNet plugin (Faust & Raes, 2016) within Cytoscape software v3.8 (Shannon et al., 2003). The calculated network was then imported to Cytoscape for visualization. Cytoscape plugin cytoHubba (Chin et al., 2014) with “degree” method was used to predict the top eight keystone nodes in the respective networks.

The metagenome-assembled genomes (MAGs) were retrieved in MetaWRAP v1.0 (Uritskiy et al., 2018) using the default parameters (–metabat2 –maxbin2 –concoct). The quality of the MAGs was estimated with CheckM v1.0 (Parks et al., 2015), and only MAGs with completeness > 50% and contamination < 10% were kept for downstream analysis. Prodigal v1.0 (Hyatt et al., 2010) (https://github.com/hyattpd/Prodigal) with default parameters was used to predict the open reading frames (ORFs) on the MAGs. The ORFs were annotated against the Kyoto Encyclopedia of Genes and Genomes (KEGG) database (Kanehisa & Goto, 2000) using eggNOG-mapper v2.0 (Cantalapiedra et al., 2021), with the default parameters (–evalue 0.001 –score 60 –pident 40 –query_cover 20 –subject_cover 20). The MAGs were submitted and annotated by GTDB-TK v1.0 for taxonomic assignment (Chaumeil et al., 2022). A combination of VIBRANT v.1.2.1 (Kieft et al., 2020) (-virome mode -l 5000) and DeepVirFinder v.1.0 (Ren et al., 2020) (score >=0.9; P value <= 0.05) were applied for viral scaffold recovery and analyses under default parameters. CheckV (v.0.8.1; database v.1.0) (Nayfach et al., 2021) was applied for quality assessment. PhaGCN v1.0 (https://github.com/KennthShang/PhaGCN) (Shang et al., 2021) was applied for virus taxonomic assignment, and PhaTYP v1.0 (https://github.com/KennthShang/PhaTYP) (Shang et al., 2023) for lifestyle classification and CHERRY v1.0 (https://github.com/KennthShang/CHERRY) (Shang & Sun, 2022) for host prediction, followed by protein sequence clustering through software Orthofinder v.1.0 (Emms & Kelly, 2019) under default procedures, as well as functional annotation against (BLASTP, E-value < 1e-5) the Prokaryotic Virus Orthologous Groups (pVOGs) database (Grazziotin et al., 2017). PhyML v1.0 (Lefort et al., 2017) under default parameters was applied for phylogenetic tree construction with Maximum Likelihood (ML) method and 1,000 bootstrap replicates (with bootstrap values in red showing on respective branches). Sequence similarity networks (SSNs) of the targeted gene families were calculated via EFI-EST Tools v1.0 under default parameters (E-value < 1e-5) (Gerlt et al., 2015).

## 3 Results

### 3.1 Change in soil physiological properties and plant growth parameters

**Figure 1:**
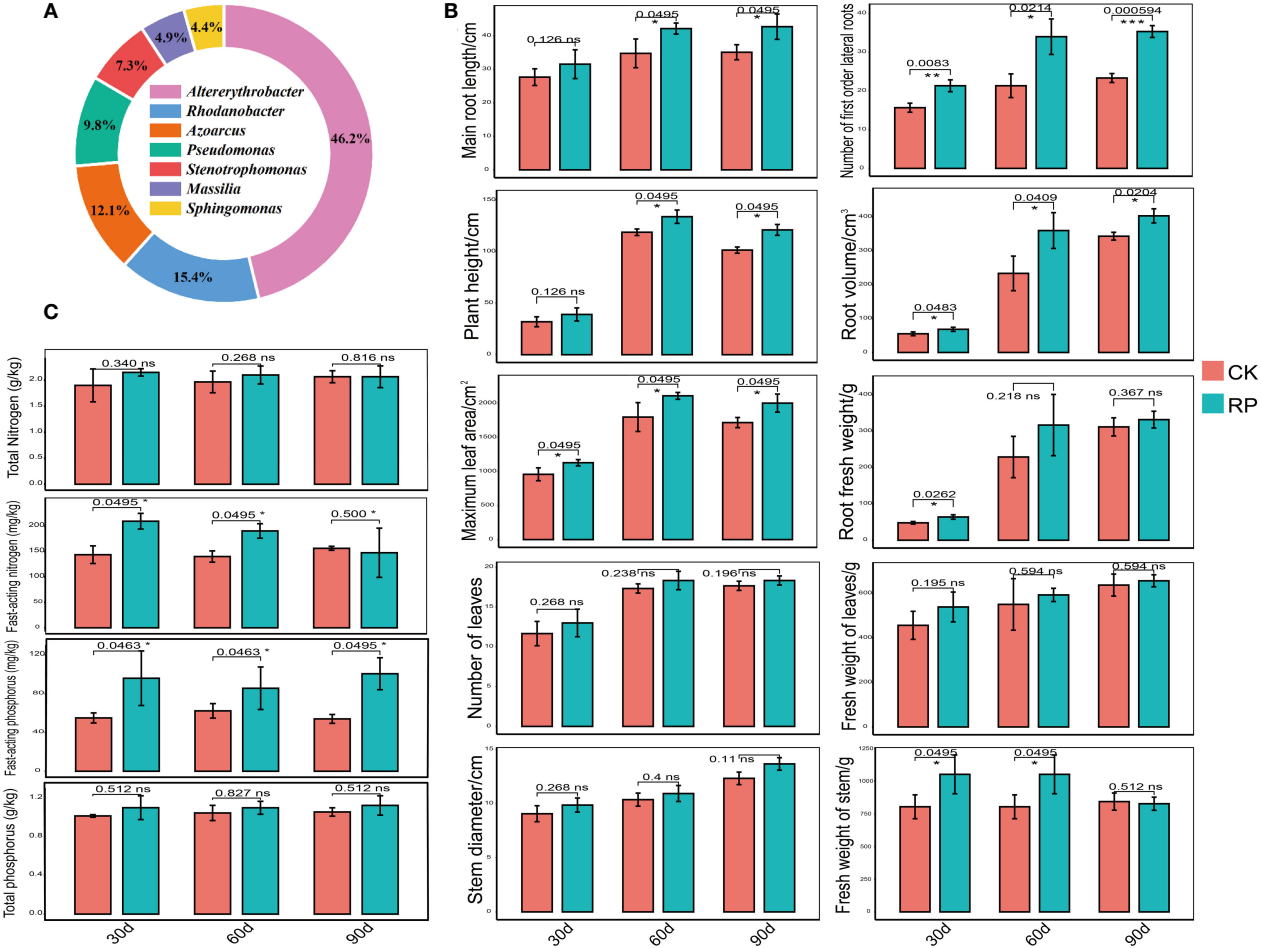
Comparisons of the physiochemical properties of soil samples and plant growth parameters of the control (CK, red color) or microbial agent treatment (RP, blue color) groups across the 90-day experimental period. **(A)** Composition of the applied functional microbial agents. **(B)** Comparisons of plant growth parameters. **(C)** Comparisons of physiochemical properties of soil samples. Three replicates were taken. Error bars represent standard error. * stands for p < 0.05 and NS, not significant.

In this study, we developed a bacterial agent composed primarily of *Altererythrobacter, Rhodanobacter, Azoarcus, Pseudomonas, Stenotrophomonas, Massilia*, and *Sphingomonas* (**Figure 1A**), identified through amplicon sequencing. These taxa have all been shown to have effects on nitrogen transformation and/or biomass promotion (Jaiswal et al., 2017; Duan et al., 2022). The control group did not receive any experimental manipulation, while the treatment group was treated with the specific microbial inoculum. We examined the effects of applying compounded biological fertilizer on soil properties over a 90-day experimental period (**Tables 1**, **2**). The results revealed that the application of the biofertilizer significantly increased the content of soil fast-acting nitrogen (AN) by 46.7% and fast-acting phosphorus (AP) by 88.6% compared to the control group (*p* < 0.05). It also positively influenced plant growth parameters, including main root length (by 23.5%), plant height (by 19.8%), maximum leaf area (by 17.3%), fresh weight of stem (by 29.9%), root fresh weight (by 38.4%), root volume (by 54.5%), and the number of first-order lateral roots (by 61.9%, **Figures 1B, C** and Supplementary Figure S1 at https://doi.org/10.6084/m9.figshare.23544930.v2). Other soil and plant growth properties have also increased but not significantly.

**Table 1:** Plant growth parameters of soil samples of the control (CK) or microbial agent treatment (RP) groups across the 90-day experimental period.

Treatment	CK-30d	CK-60d	CK-90d	RP-30d	RP-60d	RP-90
Main root length/cm	27.70 ± 2.03a	34.8 ± 3.50b	35.13 ± 1.84b	31.57 ± 3.51a	42.20 ± 1.35a	42.77 ± 3.09a
Root volume/cm^3^	55.13 ± 4.51b	233.50 ± 41.73b	343.33 ± 9.43b	68.40 ± 4.91a	360.00 ± 43.20a	403.33 ± 17.00a
Number of first order lateral roots	15.67 ± 0.94b	21.33 ± 2.49b	23.33 ± 0.94b	21.33 ± 1.25a	34.00 ± 3.74a	35.33 ± 1.25a
Root fresh weight/g	47.99 ± 2.97b	229.1 ± 46.41a	312 ± 20.43a	63.99 ± 5.01a	317.03 ± 68.81a	332 ± 18.87a
Fresh weight of leaves/g	456.69 ± 51.46a	550.87 ± 94.58a	637.6 ± 40.32a	539.24 ± 54.69a	593.40 ± 24.35a	656.83 ± 21.99a
Fresh weight of stem/g	803.13 ± 44.49a	809.63 ± 78.24a	842.8 ± 53.43a	1030.87 ± 130.48a	1051.46 ± 120.44a	826.30 ± 40.98a
Plant height/cm	31.93 ± 3.95a	118.83 ± 2.58b	101.5 ± 2.45b	39.03 ± 5.20a	133.93 ± 5.29a	121.10 ± 4.32a
Stem diameter/cm	9.07 ± 0.60a	10.37 ± 0.49a	12.33 ± 0.47b	9.87 ± 0.53a	10.93 ± 0.60a	13.67 ± 0.47a
Number of leaves	11.67 ± 1.25a	17.33 ± 0.47a	17.67 ± 0.47a	13.00 ± 1.41a	18.33 ± 0.94a	18.33 ± 0.47a
Maximum leaf area/cm^2^	959.26 ± 77.32b	1800.70 ± 173.13a	1719.93 ± 60.91b	1129.18 ± 37.40a	2112.32 ± 40.96a	2005.33 ± 108.67a

a Data are presented as the mean ± standard error of the mean for three replicates.

**Table 2:** Physiochemical properties of soil samples of the control (CK) or microbial agent treatment (RP) groups across the 90-day experimental period.

Treatment	CK-30d	CK-60d	CK-90d	RP-30d	RP-60d	RP-90d
Water content %	21.94 ± 4.19a	24.97 ± 0.59a	21.70 ± 4.93a	23.25 ± 2.58a	21.07 ± 1.84a	22.88 ± 3.62a
pH	6.00 ± 0.20ab	5.70 ± 0.10ab	5.90 ± 0.10a	5.70 ± 0.17ab	5.57 ± 0.25b	5.77 ± 0.21ab
Organic matter (g/kg)	29.79 ± 0.86a	32.97 ± 0.20a	31.87 ± 1.06a	32.25 ± 2.59a	32.11 ± 3.38a	31.40 ± 1.40a
Total Nitrogen (g/kg)	1.82 ± 0.36a	1.95 ± 0.19a	2.08 ± 0.14a	2.10 ± 0.05a	2.10 ± 0.18a	2.08 ± 0.20a
Fast-acting nitrogen (mg/kg)	143.97 ± 17.40b	140.36 ± 11.11b	156.55 ± 3.88b	209.86 ± 15.57a	180.75 ± 31.35ab	147.76 ± 48.42b
Effective phosphorus (mg/kg)	55.05 ± 5.09bc	62.13 ± 7.75bc	53.78 ± 4.52c	96.37 ± 28.09a	85.85 ± 21.75ab	100.96 ± 16.74a
Fast-acting potassium (mg/kg)	850.00 ± 416.08a	816.67 ± 38.19a	933.33 ± 344.90a	1100.00 ± 139.19a	1225.00 ± 163.94a	991.67 ± 190.94a
Total phosphorus (g/kg)	1.02 ± 0.01a	1.05 ± 0.08a	1.06 ± 0.04a	1.10 ± 0.12a	1.10 ± 0.07a	1.12 ± 0.10a
Total potassium (g/kg)	11.99 ± 0.53b	11.94 ± 0.69b	12.22 ± 0.18ab	12.43 ± 0.40ab	12.92 ± 0.47a	11.96 ± 0.14b

a Data are presented as the mean ± standard error of the mean for three replicates.

### 3.2 Changes in soil microbial community structure

**Figure 2:**
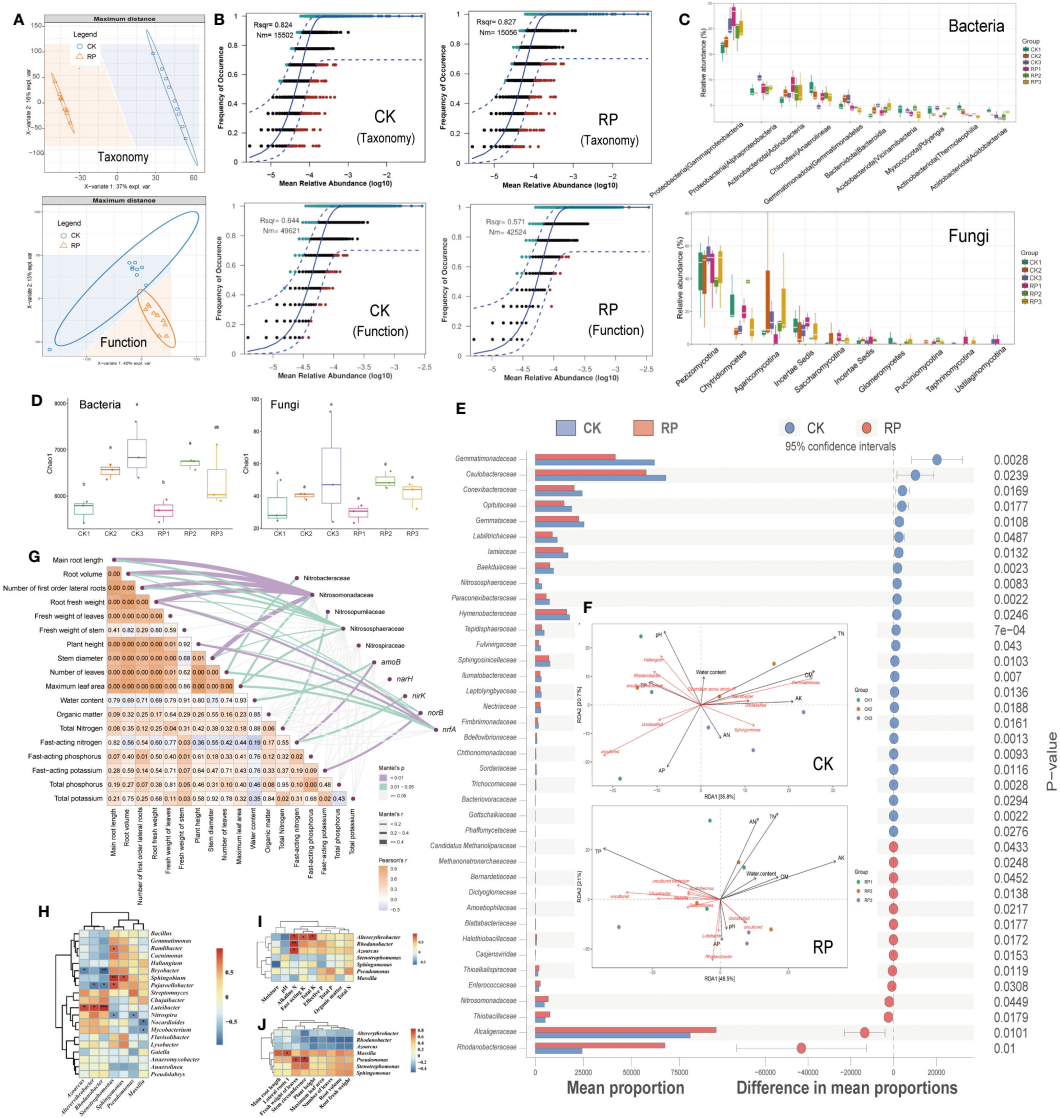
Soil microbial community composition and distribution traits in the control (CK, red color) or microbial agent treatment (RP, blue color) groups and the effect of the applied functional microorganisms. **(A)** Partial least squares discriminant analysis (PLS-DA) scores plot of microbial taxonomic and functional gene cluster profiles in the control and treatment groups (CK and RP). **(B)** Fit of the neutral community model (NCM) of the assembly process of microbial community taxonomic (top) and functional profiles (bottom), respectively. The orthogroups more frequently present than predicted are in cyan, whereas those less frequently are in red. The blue dashed lines represent 95% confidence intervals around the model prediction and the orthogroups fallen into the confidence intervals are regarded as neutrally distributed. Nm indicates that the values of the meta-community size times immigration, Rsqr indicates the fit to the neutral model. Neutral processes are the part within 95% confidence interval (red) while non-neutral are the parts including above and below prediction (dark green). **(C)** Bacterial and fungal community composition at the class level. Error bars represent standard error. **(D)** Alpha diversity analysis between control and treatment groups at 30, 60 and 90 days (corresponding to ck1-3 and rp1-3 in the labels). **(E)** Statistically significant differences in the abundance of microbial taxa between control and treatment groups analyzed by the STAMP software. **(F)** Redundancy analysis (RDA) showing the relationship between composition of the soil microbial community and physiochemical properties of soil samples factors in the control (CK, top) and treatment (RP, bottom) groups at 30, 60 and 90 days (corresponding to CK1-3 and RP1-3 in the labels). **(G)** Correlations were shown by the depth of colors, the significance showed with numbers, which were determined by the Mantel tests. **(H)** Correlation between functional microorganisms and soil microorganisms. **(I)** Correlation between functional microorganisms and soil physicochemical properties. **(J)** Correlation between functional microorganisms and crop characteristics. Single and double asterisks indicate statistical significance at P<0.05 and P<0.01, respectively.

Partial least squares discriminant analysis (PLS-DA) revealed that the microbial composition and functional gene cluster profiles in the control and treatment groups, designated as CK and RP respectively, formed distinct clusters (**Figure 2A**), indicating distinct community compositions (ANOSIM, *p <*0.001). The neutral community model (NCM) simulated a significant portion of the correlation between the occurrence frequency of microbial taxa and their relative abundance (**Figure 2B**, top), explaining 82.4% and 82.7% of the community variance for CK and RP groups, respectively. This assertion is further supported by prior research which suggests that the formation of microbial communities in agricultural soils is primarily influenced by neutral (stochastic) processes, in contrast to other natural environments such as grasslands. This is because human activities and the ongoing cultivation of agricultural land result in the continuous input of plant litter and root exudates, which serve as abundant sources of nutrition for soil-borne microbes. As a result, the stochastic influx and dispersal of these microbes are enhanced (Goss-Souza et al., 2017; Jiao et al., 2021). Conversely, the occurrence frequency of microbial functional profiles and their relative abundances were simulated to a lesser extent by NCM, contributing to 64.4% and 57.1% for CK and RP groups, respectively (**Figure 2B**, bottom), lower than that of microbial taxa composition. This implies that the microbial function is subject to fewer stochastic processes. In other words, the environment may select for functional genes rather than species, supporting previous studies on global nitrogen-cycling microbes (Song et al., 2022). Furthermore, the application of the bacterial agent decreased the NCM fit (Rsqr) by 12% and the predicted immigration rate (Nm-value) by 15% in the NCM of functional gene profiles. This suggests that the bacterial agent may have increased the deterministic effect on functional genes. However, this impact was less pronounced concerning the microbial composition.

Before and after the addition of microbial agents, the dominant soil bacteria at the phylum level were mainly Proteobacteria, Chloroflexi, Actinobacteriota and Acidobacteriota, and the dominant fungi were mainly Dikarya, Chytridiomycota and Mucoromycota (Supplementary Figure S2 at https://doi.org/10.6084/m9.figshare.23544930.v2); at the class level, the soil bacteria community was dominated by Gammaproteobacteria, Alphaproteobacteria, Actinobacteria, Anaerolineae and Gemmatimonadetes, and the dominant fungi *were all mainly Pezizomycotina Chytridiomycetes, and Agaricomycotina* (**Figure 2C**); at the genus level, the dominant soil bacteria were *Haliangium, Gemmatimonas, Sphingomonas, Rhodanobacter* and *Streptomyces*, and the dominant fungi were mainly Mortierella, Aspergillaceae, Coniochaetaceae, Chaetomiaceae and Ophiocordycipitaceae (Supplementary Figure S3 at https://doi.org/10.6084/m9.figshare.23544930.v2). Alpha diversity analysis between control and treatment groups at 30, 60 and 90 days showed that soil bacterial diversity increased significantly (*p*<0.05) from day 30 to day 60 in both groups, and continued to increase at day 90 in the control group, while it decreased at day 90 in the microbial treatment group (**Figure 2D**). At family level, the relative abundance of some taxa in treatment group was significantly (*p*<0.05) higher than that in control group, including the nitrogen turnover of Nitrosomonadaceae, Alcaligenaceae, and Rhodanobacteraceae, the methanotrophs of *Candidatus* Methanoliparaceae, Methanonatronarchaeaceae, and the putative sulfur oxidizers of Halothiobacillaceae, Thioalkalispiraceae, and Thiobacillaceae. Among them, the methanotrophs have an impact on soil nitrogen source content by performing the nitrite-dependent methane oxidation(Mosley et al., 2022; Wang et al., 2023). Meanwhile, the microbes possessing the abilities to oxidize sulfur compounds into plant utilizable sulfate form (SO_4_^2-^) may also boost plant growth (Chaudhary et al., 2023). In comparison, other taxa such as Gemmatimonadaceae and Gemmataceae were relatively depleted (*p*<0.05) in the RP group (**Figure 2E**). Consistent with this, the relative abundance of the dominant bacterial genra, *Streptomyces* and *Rhodanobacter* were found to increase significantly after the addition of microbial agent at 30 days, and then the relative abundance of *Streptomyces* continued to increase steadily at 60 and 90 days (Supplementary Figure S4 at https://doi.org/10.6084/m9.figshare.23544930.v2). In comparison, the relative abundance of the dominant bacterial genus, such as *Sphingomonas* and *Gemmatimonas*, were significantly decreased after 30 days of microbe agent addition. Regarding fungi, the relative abundance of the dominant fungi Aspergillaceae and Mortierella increased sharply after 30 days of microbe agent addition, and then decreased slightly at 60 and 90 days. The dominant fungal genera Coniochaetaceae, Chaetomiaceae and Ophiocordycipitaceae were significantly reduced after 30 to 90 days of microbe agent addition (Supplementary Figure S4 at https://doi.org/10.6084/m9.figshare.23544930.v2).

Redundancy analysis (RDA) showed that axes RDA1 and RDA2 explained 56.5% and 69.5% of the variation in the composition of the microbial community in the control and treatment groups, respectively (**Figure 2F**, top for control and bottom for treatment). In the control group (CK), the most influential factors on the microbial community were total nitrogen content (TN), organic matter content (OM), pH, fast-acting potassium content (AK), and fast-acting phosphorus content (AP). In contrast, in the treatment group (RP), the most influential factors were total phosphorus content, fast-acting potassium, and total nitrogen content. These results suggest that the correlations between total nitrogen content and microbial community compositions decreased after the addition of bacterial agents, indicating a decreased nitrogen dependence in the microbial community. Additionally, TP, AP showed strong negative correlation with TN. It is assumed that elevated nitrogen (N) deposition can alter the composition and effectiveness of soil phosphorus (P) and increases P limitation (Chen et al., 2018). Microbial communities have varying requirements for organic matter (OM), nitrogen (N), and phosphorus (P). High levels of these nutrients can accelerate the metabolism of certain species and lead to the production of excessive intracellular free radicals, inhibiting their growth and reproduction (Kou et al., 2023). Moreover, the pH level was found to have a strong negative correlation with fast-acting nitrogen content (AN). Acidification can significantly impact ammonium consumption, as the pH level can shift the nitrogen equilibrium. High environmental pH may lead to higher NH_3_ volatilization (Mandal et al., 2016) and lower net nitrogen mineralization rate (Li et al., 2019) in soils. OM showed strong positive correlations with AN and TN contents in both CK and RP groups. Microbial denitrification is typically linked to dissolved organic carbon concentrations (Rivett et al., 2008), and soil biomass is supposed to be a key factor driving nitrogen immobilization rate (NIR), as factors like pH mostly affect NIR indirectly by affecting soil biomass (Li et al., 2021). In the CK group, the microorganisms significantly associated with total nitrogen included *Gemmatimonas* and *Ramlibacter*. *Gemmatimonas* is a major player in the nitrogen cycle with the ability to reduce NO_2_^-^, N_2_O to N_2_ and is a key microorganism responsible for soil nitrogen loss (Lin et al., 2019). While in the treatment group (RP), fewer microorganisms were significantly associated with total nitrogen, other microbes were associated more so with total phosphorus, such as *Masilia*. These results implied that the introduction of functional bacteria may have inhibited the growth of denitrifying bacteria and influenced the correlation between microbial communities and nutrients such as soil nitrogen, phosphorus and potassium, eventually regulating the soil nutrient contents. The partial Mantel tests were used to explore the relationships between soil/plant growth parameters and the key nitrogen transformation related microbes/genes. The composition of ammonia oxidizers such as Nitrososphaeraceae and Nitrosomonadaceae were shown to significantly correlate with the main root length, root volume, number of first order lateral roots, root fresh weight and stem diameter (*r* > 0.4, *p* < 0.01) (**Figure 2G**). Nitrosopumilaceae was significantly correlated with total nitrogen (*r *> 0.2, *p* < 0.01). The *nrfA* gene abundance was significantly correlated with root fresh weight and plant height (r > 0.4, p < 0.01). Thus, the nitrogen transformation related microbes/genes could be a primary factor that affecting the soil/plant growth properties.

### 3.3 Colonization patterns of the functional microorganisms in bacterial agents

We further investigated the colonization patterns of the inoculated functional microorganisms (**Figure 1A**) at different fertility stages of soil fertility and their effects on nitrogen utilization to better understand their impact on soil traits and crop growth. The relative abundance of functional microorganisms was examined in during the plant growth period. Although the *Altererythrobacter* relative abundance was fairly consistent in the control group (CK) throughout the growth cycle, the treatment group (RP) showed an increase in *Altererythrobacter* relative abundance on 30 and 60 days compared to the control group (Supplementary Figure S5 at https://doi.org/10.6084/m9.figshare.23544930.v2). However, by 90 day, the relative abundance of *Altererythrobacter* in the RP group had decreased to a level below that of the control group at the same stage. These findings suggest that *Altererythrobacter* plays a significant role at the regrowth and vigorous stages (30 and 60 days) of the tested crop. The relative abundance of *Azoarcus* increased gradually in the control group but decreased gradually in the treatment group throughout the growth cycle (Supplementary Figure S6 at https://doi.org/10.6084/m9.figshare.23544930.v2). Notably, the difference in the abundance of *Azoarcus* between the treatment and control groups at the clumping stage (0.27%, 30 days) decreased at the peak stage (60 days) and at the picking stage (dropped further to -0.03%, 90 days). These findings suggested that after colonizing and interacting with the bacterial natives, the content of *Azoarcus* decreased gradually due to competitions with indigenous microorganisms or its poor adaptation to challenging environmental conditions. The relative abundance of the functional microorganism *Massilia* in the treatment group increased across the crop growth periods while *Massilia* decreased in the control group (Supplementary Figure S7 at https://doi.org/10.6084/m9.figshare.23544930.v2). The colonization capacity of *Massilia* increased gradually, reaching a difference in the abundance between the treatment and control groups of 0.42% in the treatment group at day 60. The stability of this functional bacterium across crop development suggests that it may play a significant ecological role in soil nutrient transformations. The abundance of *Pseudomonas* gradually increased in the CK group but fluctuated in the RP group (Supplementary Figure S8 at https://doi.org/10.6084/m9.figshare.23544930.v2). *Pseudomonas* decreased initially before increasing again. The colonization capacity of *Pseudomonas* exhibited a similar pattern with a decrease followed by an increase. Notably, the colonization capacity at day 90 was lower than that at day 30. These findings suggest that *Pseudomonas* is more active during the early resettling stage and the late picking stage of crop development. The relative content of the functional microorganism *Rhodanobacter* in both groups remain steady along the crop development (Supplementary Figure S9 at https://doi.org/10.6084/m9.figshare.23544930.v2). The abundance of the functional microbe *Sphingomonas* changed over time. In the CK group, *Sphingomonas* decreased slightly before increasing during the peak growing period (day 60). In contrast, the RP group showed an increase followed by a decrease over the same period. However, during the peak stage, the amount and colonization capacity of *Sphingomonas* significantly increased, which might be helpful to meet the elevated nitrogen demand of the crop (Supplementary Figure S10 at https://doi.org/10.6084/m9.figshare.23544930.v2).

### 3.4 Correlations between the functional microorganisms and environmental factors

In order to clarify the different changes in colonization capacity exhibited by functional microorganisms at different periods of vegetative development, we further analyzed the correlation between functional microorganisms (**Figures 2H**). The functional microorganism *Azoarcus* was positively correlated with *Luteibacter* (*r* = 0.6, *p* < 0.05) and negatively correlated with *Bryobacter* (*r* = -0.5, *p* < 0.05). *Altererythrobacter* was positively correlated with *Luteibacter* (*r* = 0.6, *p* < 0.05) and negatively correlated with *Pajaroellobacter*. *Rhodanobacter* was significantly positively correlated with *Luteibacter* and negatively correlated with *Bryobacter* and *Pajaroellobacter* (all *r* > 0.5, all *p* < 0.05). Consistent with the above-mentioned functional microbial colonization, *Azoarcus* and *Altererythrobacter* showed similar colonization patterns, while *Rhodanobacter* differed markedly from them and may be related to fluctuations of the contents of indigenous microorganisms such as *Luteibacter*, *Bryobacter* and *Pajaroellobacter*. In addition, *Stenotrophomonas* was positively correlated with *Ramlibacter*, *Sphingobium* and *Pajaroellobacter* and negatively correlated with *Nitrospira*.

The correlation between functional microorganisms and soil physicochemical parameters of tobacco planting is also analyzed (**Figures 2I, J**). *Altererythrobacter, Rhodanobacter* and *Azoarcus* all showed a significant positive correlation with soil nitrogen (all *p*-value < 0.05, respectively). The fast-acting nitrogen includes both inorganic nitrogen and organic nitrogen that has a simple structure and can be directly absorbed and used by the crop. The amount of fast-acting nitrogen can be significantly affected by the microbial metabolism in the soils (Kuypers et al., 2018; Li et al., 2019). The results suggested that our functional microorganisms, including *Altererythrobacter, Rhodanobacter* and *Azoarcus*, could enhance soil nitrogen transformation and increase soil nitrogen levels. In addition, *Altererythrobacter* was positively correlated with total and fast-acting potassium content (*r* = 0.6, *p* < 0.05), indicating that *Altererythrobacter* has the ability to dissolve potassium compounds in addition to its nitrogen transformation capacity. The correlation between functional microorganisms and the growth of crop is shown in **Figure 2J**, which shows that the number of primary lateral roots was positively correlated with *Massilia*, while *Pseudomonas* was positively correlated with leaf weight and stem circumference.

### 3.5 Key microorganisms and functional genes for nitrogen transformation

**Figure 3:**
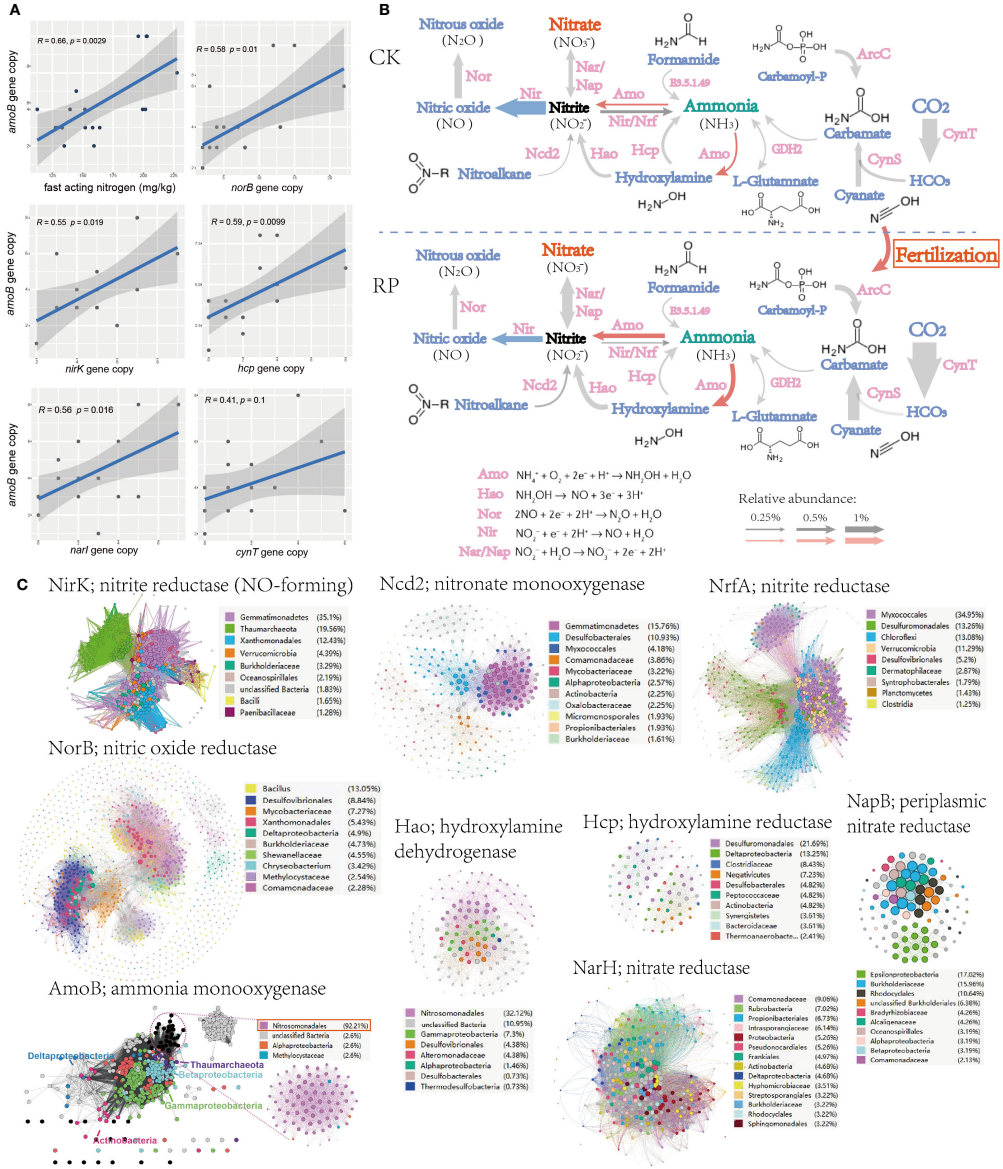
Microorganisms and functional genes for nitrogen transformation. **(A)** Linear regression analysis (spearman) also among the soil fast-acting nitrogen content and nitrogen transforming genes. **(B)** The nitrogen cycling gene abundance (showed by the size of arrow), functional categories in the control (CK) or microbial agent treatment (RP) groups depicted through metagenome sequencing. **(C)** Sequence similarity networks (SSN) of nitrogen transformation genes retrieved from the metagenomes with nodes colored by the microbial taxa encoding the gene and the node size reflecting node degree.

To identify the potential mechanism of improving soil nutrient level and plant growth, changes. The key functional genes within the microbial community were then analyzed through metagenome sequencing to investigate the mechanisms behind the raise of nutrients, especially those genes responsible for ammonium oxidation, denitrification and phosphorus solubilization. Linear regression analysis also showed that the fast-acting nitrogen content was significantly positively correlated with ammonia oxidation gene (ammonium monooxygenase, *amoB*) abundance in metagenomes (*r* = 0.66, *p* < 0.01) (**Figure 3A**). Ammonium monooxygenase have dominant effects on soil ammonia oxidation (Liu et al., 2017). Furthermore, the *amoB* gene abundance was also significantly positively correlated with genes *nirK, narI, norB, hcp*, and *cynT* (*r* ranged from 0.41 to 0.59, all *p* < 0.05, **Figure 3A**), which indicates a potential link between the ammonia-oxidizing microorganisms and other nitrogen-cycling processes in the environment. *nirK, narI, norB* are known as functional genes involved in denitrification, which convert nitrate into nitrite or nitrous oxide, leading to the loss of bioavailable nitrogen from the ecosystem. Specially, the denitrifiers harboring *nirK* gene was reported to be involved in regulating both ammonia oxidization and denitrification (Humbert et al., 2010), and the denitrifiers can also relieve the inhibition of organic carbons and decrease the toxicity of NO_2_^−^ for ammonia-oxidizing microorganisms. The positive correlations with *amoB* gene suggest that there may be an interdependence between nitrification and denitrification processes to maintain the nitrogen balance and biogeochemical cycling in soils. The metagenome analysis further indicated that the genes for ammonia oxidization and nitrification processes, and hydroxylamine dehydrogenase (*hao*) showed higher abundance after the bacterial agent application, whereas denitrification genes (e.g., *nir*) showed decrease abundance (**Figure 3B**), leading to the accumulation of fast-acting nitrate in soil and reduced gaseous nitrogen release, which can be readily taken up by plants for growth. Ammonium is oxidized to hydroxylamine by AMO, hydroxylamine to nitrite by HAO (Soler-Jofra et al., 2021). Ammonia oxidation instead of denitrification may be the main pathway for the transformation and mineralization of nitrogen.

The sequence similarity networks (SSN) of nitrogen transformation genes retrieved from the metagenomes were subsequently analyzed (**Figure 3C**). Gemmatimonadetes was found to occupy the most nodes in the SSNs of nitrite reductase (NirK) and nitronate monooxygenase (Ncd2). The degree and betweenness of Gemmatimonadetes nodes are also significantly higher than other taxa, which indicated that Gemmatimonadetes is the core taxon within respective SSN and may play essential roles in the denitrification processes. Another frequently present taxon is Nitrosomonadales, which dominates SSN nodes of ammonia monooxygenase (AmoB) and hydroxylamine dehydrogenase (Hao), indicating that Nitrosomonadales is an important ammonia oxidizer in the soil ecosystem. As mention before, the application of our bacterial agent has significantly reduced the abundance of Gemmatimonadetes while enhanced the enrichment of Nitrosomonadales (*p*<0.05, **Figure 2E**), which is also consistent with the increase abundance of nitrification and decrease of denitrification genes (**Figure 3B**).

**Figure 4:**
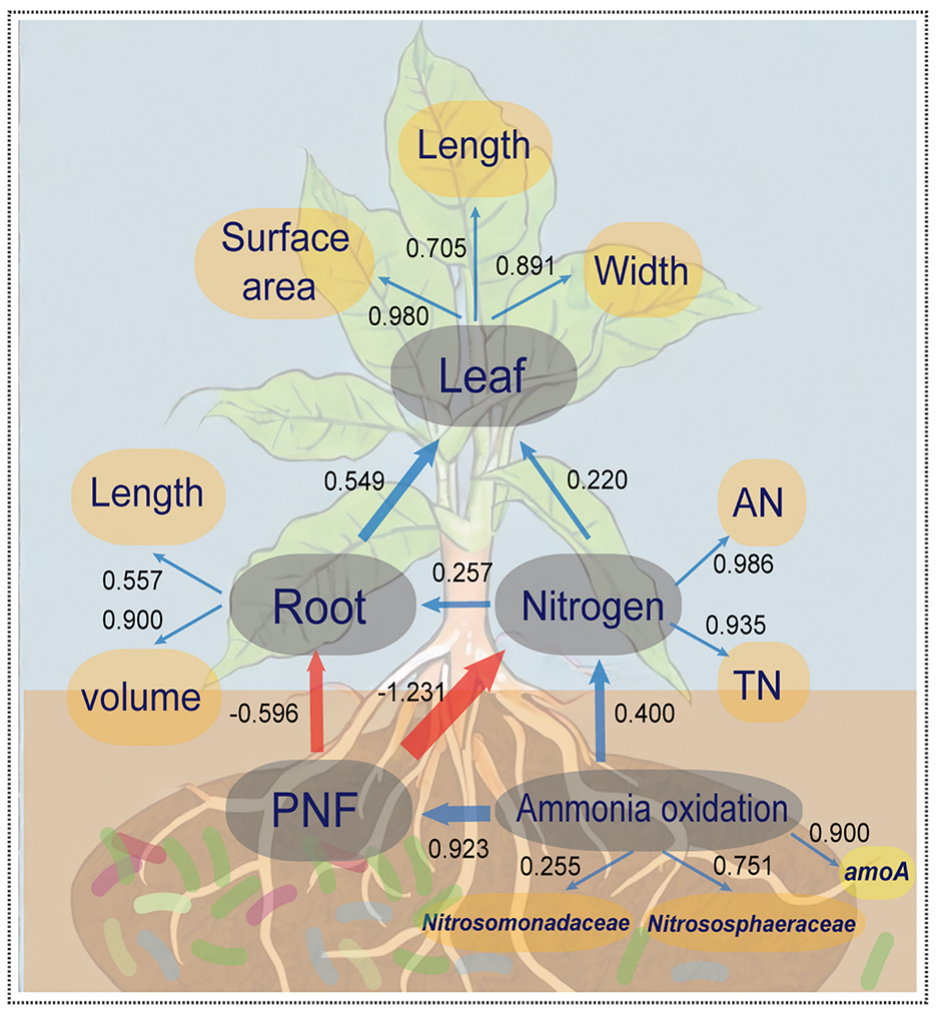
The partial least squares path model (PLS-PM) for illustrating the coupling effect of the tripartite: N-cycling bacteria, soil properties and crop growth. The yellow elliptical variables are independent variables and the grey elliptical variables are hidden variables. The blue arrows between hidden variables represent positive correlations (*p*<0.05), red arrows represent negative correlations (*p*<0.05), and the arrow thicknesses and values represent effect sizes. Values between the independent and hidden variables represent the constraints of independent variables on hidden variables.

Partial least squares path model (PLS-PM) was utilized to investigate the relationship between microbial community composition in the rhizosphere, abundance of key genes, soil nitrogen, and agronomic data on plant growth (see **Figure 4**). The overall model fit was 0.709, indicating its reliability and establishing a significant positive correlation between inter-root bacterial ammonia oxidation function and the abundance of ammonia oxidation gene *amoA*, as well as Nitrosomonadaceae and Nitrososphaeraceae. Microbial ammonia oxidation played a substantial role in the potential nitrification potential and nitrogen content in the soil. However, the potential nitrification potential (PNF) exacerbated the conversion of other nitrogen forms to nitrate, which increased the risk of loss via leaching. This directly inhibited the growth of root volume and length, indirectly causing a reduction in the maximum leaf area, length, and width of crop leaves. Consequently, these findings suggest that the addition of biofertilizer to the rhizosphere soil and regulation of ammonia-oxidizing microbe abundance in the rhizosphere microbial community are essential strategies for enhancing nitrogen utilization efficiency in crops.

### 3.6 Molecular ecological networks reveal enhanced microbial interaction

**Figure 5:**
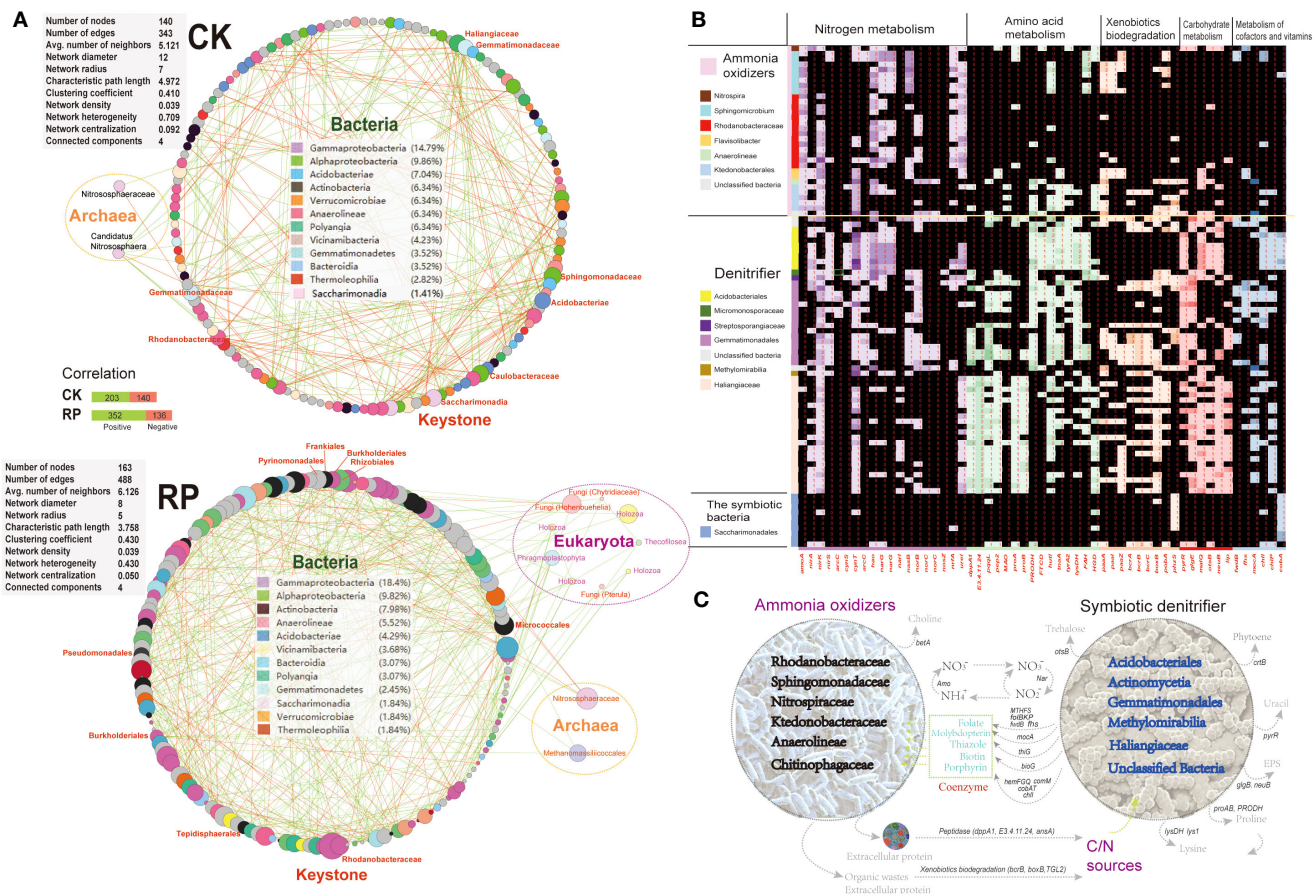
Analysis on the putative functions of keystone microorganisms. **(A)** Molecular ecological networks of microbial community in the control (CK) or microbial agent treatment (RP) groups visualized with circular layout in Cytoscape software. The links between the nodes indicate strong (*cor >*0.8) and significant (*p* < 0.05) correlations. The identified keystone hubs by cytoHubba are marked with red-color labels. **(B)** Heatmap showing distribution of gene families related with nitrogen metabolism, amino acid metabolism, xenobiotics biodegradation, carbohydrate metabolism, and metabolism of cofactors among the recovered MAGs. The gene copy numbers are correlated with the depth of cell colors. **(C)** A conceptual model of the ecological roles conducted by the microbial ammonia oxidizers and symbiotic denitrifiers in rhizosphere soils based on the annotated functions.

Molecular ecological networks (MENs) were constructed to investigate the impact of microbial biofertilizer application on microbial interactions (see **Figure 5A**). The results revealed that the complexity of the MEN increased following the application of the bacterial biofertilizer (RP group) compared to the control group (CK group). Specifically, the number of nodes increased from 140 in the CK group to 163 in the RP group. Likewise, the number of links increased from 343 in the CK group to 488 in the RP group, with a higher proportion of positive correlations observed (72.1% in RP versus 59.1% in CK). The network parameters also showed increases, including the average number of neighbors (from 5.1 to 6.1) and the clustering coefficient (from 0.41 to 0.43), suggesting a greater complexity of the MEN after biofertilizer application. The mutual relationships between bacteria and fungi were more prevalent in the MEN of RP than in CK (92.8% positive associations) (**Figure 5A**). Specifically, plant growth promotion bacteria (PGPB) including Edaphobaculum, Cyanobacteriia, Cytophaga, and Myxococcota, as well as fungal taxa Hohenbuehelia, Chytridiaceae, and Pterula, show significant correlations with fungal and bacterial taxa, respectively. Hohenbuehelia has been reported to show good growth inhibition of pathogens (Bala et al., 2011).

### 3.7 The function potentials of keystone in ecological network

The metagenome contigs were binned to the draft genomes, resulting in the recovery of 99 draft genomes with completeness above 50% and contamination lower than 10%. The majority of metagenome-assembled genomes (MAGs) belong to keystone taxa identified in the molecular ecological networks mentioned above. This includes eight MAGs from the Sphingomonadaceae family, eight MAGs from the Acidobacteria phylum, 20 MAGs from the Gemmatimonadales order, 14 MAGs from the Rhodanobacteraceae family, and 10 MAGs from the Saccharimonadia phylum. We then investigated the functional differences among each MAG to identify enriched gene functionalities. We categorized the MAGs based on the presence or absence of ammonia oxidation genes (e.g., ammonia monooxygenase, amoA) as putative ammonia-oxidizing microorganisms (e.g., Nitrospiraceae), specialized denitrifying microorganisms (e.g., Gemmatimonadales), or other symbiotic bacteria. The MAGs were found to possess a prevalence of nitrite cycling genes (e.g., ammonia oxidation, denitrification, dissimilatory nitrate reduction to ammonium), indicating that nitrogen cycling is a core function of the soil microbial communities under study. Ammonia-oxidizing and denitrifying microorganisms may play dual roles in soil nitrogen transformation. The nitrate generated from nitrification reactions can be reduced by denitrifiers to nitrite and ammonia, which can then be utilized by symbiotic microbes in the consortia, thereby increasing nitrogen turnover efficiency. Importantly, these MAGs also exhibited complementary metabolic processes, suggesting the occurrence of cross-feeding on essential metabolites (**Figures 5B, C**). Specifically, symbiotic denitrifier MAGs were enriched in key genes for cofactor and vitamin biosynthesis required for oxidative phosphorylation and the CoA pathway. These included molybdopterin (*mocA*), folate (*fhs, MTHFS, folBKP*, and *fwdB*), thiazole (*thiG*), biotin (*bioG*), and porphyrin (*hemFGQ, comM, cobAT* and *chlI*), which were all depleted in the recovered ammonia oxidizer MAGs. Coenzymes and amino acids were identified as significant metabolic mediators that influence the composition of microbe communities. Molybdopterin and folate serve as cofactors for many oxidoreductases, such as the formate dehydrogenases involved in the acetyl-coA production and CO_2_ fixation (Cotton et al., 2018). These secondary metabolites are likely crucial for the growth and activity of ammonia-oxidizing microbes, as they affect core metabolisms (Wang et al., 2016). Other secondary metabolites, such as the biotin cofactors, have been also shown to be cross-fed among nitrogen-transforming consortia members (Seth & Taga, 2014) due to their energy-intensive synthesis. In a previous study, the development and activity of microbes lacking folate were significantly improved when co-cultured with microbes that produce folate (Zhou et al., 2011). The symbiotic microbes enriched in cofactor synthesis abilities may have a reciprocal interaction with ammonia-oxidizing microbes. Furthermore, several key genes involved in the synthesis of crucial amino acids were absent in the MAGs of ammonia oxidizers but enriched in the symbiotic denitrifiers. These include genes for the production of proline (*proAB, PRODH*) and lysine (*lys1, lysDH*), suggesting that the ammonia oxidizers rely on acquiring essential nutrients from other microorganisms for their growth. Amino acid cross-feeding has been reported as a defensive mechanism of ammonia-oxidizing consortia under stressful conditions (Zhao et al., 2018).

Symbiotic denitrifying microorganisms possess several additional types of peptidases, such as those encoded by *dppA1, E3.4.11.24*, and *ansA*. These peptidases enable them to hydrolyze proteins and polypeptides more efficiently than ammonia-oxidizers (**Figures 5B, C**). Also, the symbiotic denitrifier MAGs harbored key genes for the biosynthesis of the nucleotide sugar (*pyrR*) and exopolysaccharide (*glgB, neuB*) that are depleted in the ammonia-oxidizing MAGs. Additionally, the symbiotic denitrifiers possess unique key genes (e.g. *bcrB, boxB, TGL2*) for the biodegradation of refractory organic matters as carbon source to support heterotrophic growth in the consortia. The symbiotic consortia also encoded complementarily the biosynthesis for antioxidant pigment phytoene (*crtB*), osmoprotectant glycine betaine (*betA*) and trehalose (*otsB*).

Despite the differences mentioned above, plant growth promotion (PGP) traits were found to be widely present and highly abundant across the MAGs (Supplementary Figure S11 at https://doi.org/10.6084/m9.figshare.23544930.v2). These traits include genes responsible for enzymatic hydrolysis of phosphorus compounds (e.g., alkaline phosphatase *phoD*, phosphatidate phosphatase *phoN*), metabolism of plant hormone (e.g., indole-3-acetate monooxygenase *iacA*, indole-3-glycerol phosphate synthase *trpC*), production of secondary metabolite acetoin (*acoRAB*), catecholate siderophore (*fiu*), osmoprotectants (e.g., *opuBDC, ectD, stfO, treAT*, and *otsB*) and sulfur metabolism (e.g. *soeA, SUOX, tmoC*).

### 3.8 Viral sequence mining from metagenome

**Figure 6:**
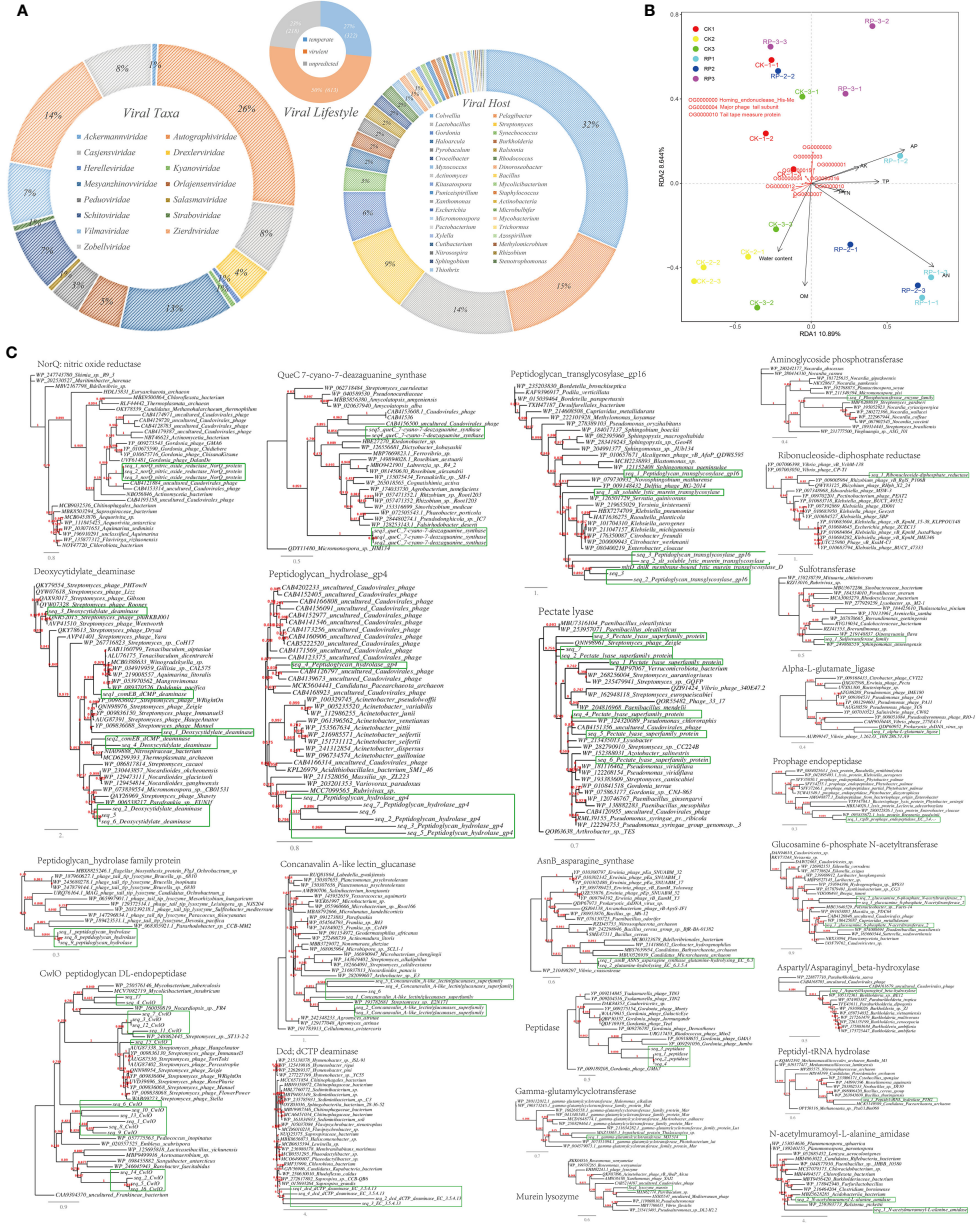
Viral sequence from metagenome and functional annotation. **(A)** Pie charts showing the viral family classification by PhaGCN, lifestyle classification by PhaTYP and host prediction by CHERRY. **(B)** Redundancy analysis (RDA) based on the environmental factors and the viral functional profiles in viromes retrieved from tested metagenomes. **(C)** Phylogenetic analyses of the representative metabolic viral genes in this study (marked with green rectangle) with the existing sequences from Genbank database (constructed with PhyML, the branch length is proportional to the number of substitutions per site).

Viruses have been found to play a significant role in shaping the composition of bacterial communities (Emerson et al., 2018). Gaining a deeper understanding of the virome in plant-associated soils could help unravel their potential roles in perturbation responses and driving microbial adaptation to diverse environments. In this study, a total of 3,589 scaffolds were examined from the soil metagenome, and it was predicted that they were viral. Among these viral genomes, 2.9% were complete, 6.9% were of high quality, and 368 were predicted to be lysogenic/temperate (**Figure 6A**). The viral scaffolds were primarily assigned to viral taxa such as Drexlerviridae, Casjensviridae, and Autographiviridae (**Figure 6A** and Supplementary Figure S12 at https://doi.org/10.6084/m9.figshare.23544930.v2). These viruses were predicted to be capable of infecting microbial hosts *Colwellia* (32%), *Pelagibacter* (15%), *Lactobacillus* (14%), *Streptomyces* (9%), *Gordonia* (6%) and known nitrogen-tranforming PGPB such as *Rhizobium, Nitrosospira, Azospirillum*, and *Burkholderia*. Redundancy analysis (RDA) based on the environmental factors and the viral functional profiles in the retrieved viromes showed that the viromes were well separated by the samples (**Figure 6B**, ANOSIM, *p* < 0.05), indicating that the viromes were sensitively responsive to changes in the environment. Furthermore, viral orthogroup encoding homing endonuclease (His-Me) and several unannotated orthogroups showed strong negative correlation with soil organic matter content (OM). Similarly, the viral orthogroup encoding the major phage tail subunit exhibited a strong negative correlation with soil fast-acting nitrogen content. These findings indicate that dominant lytic bacteriophages could serve as significant driving forces by infecting and killing soil microorganisms, including those involved in nitrogen cycling, leading to a decrease in soil biomass and uncontrolled nitrogen loss.

Interestingly, the viral scaffolds contained numerous genes that are involved in the transformation of carbon, nitrogen, sulfur, and phosphorous nutrients, suggesting that viruses may enhance the cycling of these nutrients through lateral gene transfer. For example, our analysis revealed genes encoded for the metabolism of nitrogen-containing compounds, such as nitric oxide reductase (NorQ), asparagine synthase (AsnB), aspartyl/asparaginyl beta-hydroxylase, alpha-L-glutamate ligase, 7-cyano-7-deazaguanine synthase (QueC), glutamylcyclotransferase, deaminase, and glucosamine 6-phosphate N-acetyltransferase. Additionally, we identified genes encoded for multiple polysaccharide hydrolases and peptidases that enable growth on residues of dead microbes, such as peptidoglycan hydrolase, prophage endopeptidase, murein lysozyme, lectin glucanase, peptidoglycan DL-endopeptidase (CwlO), and peptidyl-tRNA hydrolase. Furthermore, the viral genes included those involved in the metabolism of phosphorus compounds, such as ribonucleoside diphosphate reductase, aminoglycoside phosphotransferase, phosphatase, phosphate kinase, as well as genes encoding sulfide-containing compounds such as thioredoxin, acyl-CoA thioesterase I (TesA), and sulfotransferase (**Figure 6C**).

## 4 Discussion

In the present study, the application of the biofertilizer resulted in a significant increase (*p* < 0.05) in soil fast-acting nitrogen (alkali hydrolyzed nitrogen) and fast-acting phosphorus contents. This observation suggests an enhanced transformation and solubilization of nitrogen and phosphorus in response to the biofertilizer application, leading to improved plant growth. Our findings align with previous studies that employed microbial agents containing *Bacillus*, *Sinorhizobium* and *Streptomyces*, which also reported significant increases in organic matter, alkali hydrolyzed nitrogen, available phosphorus, available potassium, as well as the activities of invertase and urease in soils compared to control groups. These studies also documented enhanced plant growth as a result of microbial agent application (Guo et al., 2019; Deng et al., 2021). Furthermore, research by Allsup et al. (2023) demonstrated that saplings inoculated with microbial communities from harsh environmental conditions exhibited higher survival rates when faced with various stresses. This phenomenon was attributed to the increased fungal diversity associated with the microbial communities. Similarly, our biofertilizer contains components that effectively colonize the soil and enrich functional microorganisms. This colonization process promotes nitrogen transformation, nitrogen retention, and plant nutrient uptake. The persistence of inoculated microorganisms in plant roots, even after three years of inoculation (Allsup et al., 2023), indicates that the inoculated microbial communities retain their properties and effects on host plants over time. This highlights the potential of pre-inoculating plants with biofertilizers as a viable method for restoring microbial communities and their functions (Allsup et al., 2023). Additionally, Zhao et al. (Zhao et al., 2021) conducted a study that focused on the effects of environmental stress, particularly heavy metal exposure, on soil nitrification, nitrogen mineralization, and associated microbial populations. Their findings revealed that heavy metal exposure initially stimulates soil nitrification, followed by a facilitative effect on nitrogen mineralization and an increase in the microbial populations involved. These insights provide valuable information regarding the interplay between soil microbes and nutrients, and they offer potential strategies for manipulating plant-associated microbiota to enhance crop yields.

The present study observed a greater complexity in the microbial ecological network (MEN) following the application of biofertilizer. This complexity can be attributed to the positive effects of microbial synergistic interactions, which enhance the adaptability of microbial consortia and broaden their ecological niches (Wang et al., 2022). Additionally, higher stress levels may lead to a prevalence of positive associations over competitive relationships (Abrego et al., 2020).

In the control group (CK), the MEN was characterized by the presence of keystones belonging to denitrifying bacteria families Gemmatimonadaceae and Haliangiaceae. However, these keystones were not identified in the MEN of the treatment group (RP). Conversely, the MEN of the RP group exhibited the presence of well-known plant growth promotion bacteria, such as Burkholderiales and Rhizobiales. These bacteria are known for their ability to promote root elongation and enhance plant nutrient uptake through nitrogen transformation and siderophore secretion (Naveed et al., 2014). These findings align with previous reports that indicate keystone microorganisms within the soil community, which play crucial ecological roles, can be particularly sensitive to environmental changes and undergo succession as conditions fluctuate (Sun et al., 2020b; Allsup et al., 2023). The MEN analysis also revealed the presence of mycorrhiza helper bacteria, including *Pseudomonas*, which have been reported to stimulate fungal spore germination, enhance mycelial growth, facilitate microbial colonization of plants, and provide protection against root pathogens (Frey-Klett et al., 2007). Furthermore, diazotrophic bacteria residing in ectomycorrhizal tissues can supply nitrogen resources to the fungal partners involved in soil symbiosis (Frey-Klett et al., 2007). Fungi, in turn, can influence soil nitrogen transformation through various mechanisms, including nitrogen transfer to plants (Veresoglou et al., 2012). These results suggest that the application of bacterial agents in this study potentially improved the cross-kingdom cooperation between bacteria and fungi.

Additionally, our study uncovered potential interactions between archaea and bacteria. The presence of ammonia-oxidizing archaea Nitrososphaeraceae (Thaumarchaea) was observed in the MENs of both CK and RP groups, along with Dehalococcoidia, Acidobacteriae, and Acidimicrobiia. This suggests the existence of collaborative relationships between ammonia-oxidizing and symbiotic denitrifying microorganisms. Previous research has demonstrated that denitrifying and ammonia-oxidizing microbial communities can form interconnected networks to withstand environmental stressors (Hernandez et al., 2021).

Moreover, the MEN analysis revealed the co-occurrence of methanotrophic archaea Methanomassiliicoccales with the ammonia oxidizer Nitrososphaeraceae, as well as bacterial taxa Thermodesulfovibrionia and Verrucomicrobiae in the RP group. Methanotrophs have the ability to oxidize methane to CO2, using methane as their sole carbon and energy source, with nitrite generated by the ammonia oxidizer serving as the electron acceptor (Ettwig et al., 2010). Additionally, methanotrophs can directly oxidize ammonia to nitrite (Holmes et al., 1995). It is noteworthy that methanotrophs and ammonia oxidizers share numerous metabolic similarities and a common evolutionary history (Stein et al., 2012).

In brief summary, the findings of this study highlight the increased complexity in the MEN after the application of biofertilizer. The observed changes in keystone microorganisms, cross-kingdom cooperation, and potential interactions between archaea and bacteria provide valuable insights into the ecological dynamics of microbial communities in response to environmental factors and agricultural interventions.

In our study, we examined the functional genes shared or differed among groups of metagenome-assembled genomes (MAGs) (see section 3.7). Based on our findings, we propose that microbial cross-feeding plays a crucial role in increasing biological variability and ecosystem resilience, thereby influencing the breadth of natural niches. These cross-feedings can also have an impact on the performance of biofertilizers.

One of the key contributions of functional microorganisms is their ability to convert refractory nutrients, such as polyphosphate or sulfuric compounds, into their ionic form, which makes them more readily available for plants to uptake. Additionally, these microorganisms can modulate the production of plant hormones, further contributing to vegetation development (Rodríguez et al., 2006; Chaudhary et al., 2023). Symbiotic denitrifiers are particularly important agents in the degradation of extracellular peptides that are primarily synthesized by autotrophs (Lawson et al., 2017). Furthermore, the accompanying microorganisms in the consortia utilize common amino acids as a carbohydrate and energy resource in the extracellular matrix (Lawson et al., 2017). The breakdown of proteins results in the production of ammonium, which can react with nitrite to stimulate the ammonia-oxidizing response (Takenaka et al., 2009).

The formation of an extracellular matrix, composed of exopolysaccharides, is crucial for the development of microbial aggregation. Symbiotic denitrifiers engage in cross-feeding on nucleotide sugars and release other macro-molecule carbohydrates, which can enhance the agglomeration and activities of ammonia-oxidizing microorganisms. Additionally, the electron generated from the biodegradation of organic matter can fuel the process of ammonia oxidation (Zhang et al., 2022). Under stressful conditions, microbial consortia have the ability to increase their biosynthesis of exopolysaccharides and their capability to aggregate (Lawson et al., 2017). Moreover, cross-feedings of osmoprotectants with high biosynthetic costs can benefit the acclimation of the symbiotic consortia (Huo et al., 2020).

In brief summary, our study provides valuable insights into the intricate interactions and processes involved in microbial cross-feeding, emphasizing its significance in enhancing biological variability, ecosystem resilience, and the performance of biofertilizers.

Finally, abundant viral contigs carrying functional genes related to nitrogen metabolism were retrieved from the soil metagenomes in current study. Phylogenetic analyses of the viral genes indicated that these sequences are distinct from known sequences but cluster with viral sequences in the GenBank database, suggesting the reliability of the predictions. The findings align with previous studies that have demonstrated the ability of viruses to regulate the carbon and nitrogen metabolism of their microbial hosts to meet their own replication and proliferation needs. For example, viruses from marine environments can carry the nitrogen assimilation regulatory gene P-II (Roux et al., 2016) and the ammonia monooxygenase gene *amoC* (Ahlgren et al., 2019). Marine cyanobacterial viruses can inhibit the host photosynthesis through the CP12 protein and divert host carbon flow to the pentose phosphate pathway as to promote their own nucleic acid synthesis (Thompson et al., 2011). Viruses also possess genes encoding glycosidic hydrolases that facilitate the degradation of plant apoplastic materials by their microbial hosts, including the mannan endonuclease gene for hemicellulose galactomannan (Emerson et al., 2018) and the polycopper oxidase gene for lignin.

Furthermore, certain viruses can modulate the uptake of extracellular nutrient resources by the host. Studies have shown that viruses stimulate extracellular nitrogen uptake, even when the host is not nutrient-limited (Cohen, 1949). Isotopic studies confirmed that the synthesis of virulence particles of *Emiliania huxleyi* and *Synechococcus* depends on extracellular carbon and nitrogen sources from the host (MaalØE & Stent, 1952; Waldbauer et al., 2019). Under nitrogen source-limited conditions, cyanobacterial viruses can splice switch elements from the inter-cluster of host nitrogen fixation genes (*nifD, fdxN*, and *hupL*) and insert them into the genome of newly formed spores (Golden et al., 1985; Henson et al., 2011), and the former parent cell will gain nitrogen fixation capacity (Feiner et al., 2015). Furthermore, genomic analysis of cyanobacteria has shown that the switch elements of each virus insert at the same nitrogen fixation homologous gene position, suggesting a specific integration event of the common ancestral virus (Carrasco et al., 1995; Henson et al., 2011).

The presence of metabolic genes in viral scaffolds implies that viruses may also contribute to biogeochemical processes in biofertilizer-amended soils. These findings may generate interest in virus-based strategies, such as virome transplantation, for manipulating microbial communities.

Overall, the utilization of the bacterial agent has the potential to influence the microbial nitrogen transformation process and increase the levels of effective nitrogen within agroecosystems. The research provides valuable insights into the microbial mechanisms underlying nutrient turnover and the promotion of plant growth through the use of biofertilizers, thus informing the development of sustainable agricultural practices.

In terms of future perspective, it is important to note that this study primarily examined changes in relative abundances of bacterial and fungal taxa and their functional differences without directly measuring specific microbial activities or gene expression. Further research is needed to establish the relationships between the observed changes and specific microbial functions based on quantitative expression data. Additionally, this study did not directly investigate the long-term effects of the bacterial agent on soil properties and plant growth over multiple seasons or under different environmental conditions. Therefore, future studies should consider long-term observations and experimental manipulations to better understand the efficacy and stability of the bacterial agent in different agroecosystems.

The findings of this study have important implications for agricultural sustainability and productivity. By enhancing nutrient availability and promoting plant growth, the use of biofertilizers can contribute to increased crop yields, reduced reliance on chemical fertilizers, and improved soil health. These positive outcomes can have significant societal and environmental impacts, such as reducing the environmental footprint of agriculture, improving food security, and promoting sustainable farming practices.

## 5 Conclusion

This study investigated the mechanisms through which a bacterial agent composed of *Altererythrobacter, Rhodanobacter, Azoarcus, Pseudomonas, Stenotrophomonas, Massilia*, and *Sphingomonas* improved soil properties and plant growth. Results showed that the bacterial agent significantly increased fast-acting nitrogen and fast-acting phosphorus in the soil, resulting in enhanced nutrient availability and improved plant growth. These findings highlight the potential of biofertilizers to enhance agricultural practices and increase productivity. Moreover, the study revealed shifts in the relative abundances of bacterial and fungal taxa following the introduction of the microbial agent. Changes in the microbial ecological networks were observed, including a shift in keystone taxa. Furthermore, the analysis of metagenome-assembled genomes (MAGs) of these keystone taxa indicated functional differences among them, highlighting the diverse capabilities involved in promoting vegetation development by enhancing nutrient availability and regulating the production of plant hormones.

## Data availability statement

The metagenome assembly sequences are deposited at National Genomics Data Center (cncb.ac.cn) under Bioproject PRJCA019293 at https://ngdc.cncb.ac.cn/bioproject/browse/PRJCA019293. Other data from the paper are available on request.

## Author contributions

LL: Conceptualization, Data curation, Formal Analysis, Writing – original draft. ZH: Investigation, Supervision, Writing – review & editing. QT: Investigation, Supervision, Validation, Writing – review & editing. GT: Writing – review & editing. JF: Writing – review & editing. YC: Writing – review & editing. YX: Writing – review & editing. SW: Writing – review & editing. QZ: Writing – review & editing. TL: Writing – review & editing. HY: Project administration, Resources, Writing – review & editing.
